# Citrin deficiency mimicking mitochondrial depletion syndrome

**DOI:** 10.1186/s12887-020-02409-x

**Published:** 2020-11-11

**Authors:** S. C. Grünert, A. Schumann, P. Freisinger, S. Rosenbaum-Fabian, M. Schmidts, A. J. Mueller, S. Beck-Wödl, T. B. Haack, H. Schneider, H. Fuchs, U. Teufel, G. Gramer, L. Hannibal, U. Spiekerkoetter

**Affiliations:** 1 Department of General Paediatrics, Adolescent Medicine and Neonatology, Medical Centre-University of Freiburg, Faculty of Medicine, Mathildenstraße 1, 79106 Freiburg, Germany; 2 Department of Paediatrics, Klinikum Reutlingen, 72764 Reutlingen, Germany; 3 Institute of Medical Genetics and Applied Genomics, University of Tübingen, Tübingen, Germany; 4 Center for Rare Diseases, University of Tübingen, 72076 Tübingen, Germany; 5 University Hospital Heidelberg, Centre for Paediatric and Adolescent Medicine, Division of Neuropaediatrics and Metabolic Medicine, Im Neuenheimer Feld 430, 69120 Heidelberg, Germany; 6 Department of General Paediatrics, Adolescent Medicine and Neonatology, Laboratory of Clinical Biochemistry and Metabolism, Medical Centre-University of Freiburg, Faculty of Medicine, Freiburg, Germany

**Keywords:** Citrin deficiency, Neonatal cholestasis, Hypoglycemia, Newborn screening, Urea cycle defect, SLC25A13

## Abstract

**Background:**

Neonatal intrahepatic cholestasis caused by citrin deficiency (CD) is a rare inborn error of metabolism due to variants in the *SLC25A13* gene encoding the calcium-binding protein citrin. Citrin is an aspartate-glutamate carrier located within the inner mitochondrial membrane.

**Case presentation:**

We report on two siblings of Romanian-Vietnamese ancestry with citrin deficiency. Patient 1 is a female who presented at age 8 weeks with cholestasis, elevated lactate levels and recurrent severe hypoglycemia. Diagnosis was made by whole exome sequencing and revealed compound heterozygosity for the frameshift variant c.852_855del, p.Met285Profs*2 and a novel deletion c.(69 + 1_70–1)_(212 + 1_231–1)del in *SLC25A13*. The girl responded well to dietary treatment with a lactose-free, MCT-enriched formula. Her younger brother (Patient 2) was born 1 year later and also found to be carrying the same gene variants. Dietary treatment from birth was able to completely prevent clinical manifestation until his current age of 4.5 months.

**Conclusions:**

As CD is a well-treatable disorder it should be ruled out early in the differential diagnosis of neonatal cholestasis. Due to the combination of hepatopathy, lactic acidosis and recurrent hypoglycemia the clinical presentation of CD may resemble hepatic mitochondrial depletion syndrome.

## Background

Citrin deficiency (CD) is an autosomal recessive inborn error of metabolism caused by variants in the *SLC25A13* gene [1–3]. Citrin is an aspartate-glutamate carrier located within the inner mitochondrial membrane and mainly expressed in the liver, kidney, heart and small intestine [4, 5]. Its role to transport aspartate from the mitochondrial matrix to the cytosol is important for several metabolic pathways including protein, nucleotide and urea synthesis as well as gluconeogenesis from lactate and the translocation of cytosolic NADH reducing equivalents into the mitochondria via the malate-aspartate shuttle [4]. While CD is relatively common in East Asian populations, especially in Japan, the incidence in Europe is extremely low [2], although CD is a pan-ethnic disease, and subjects have been reported from different ethnicities [6–10]. Three age-dependent clinical phenotypes are associated with CD, namely 1) neonatal intrahepatic cholestasis caused by citrin deficiency (NICCD) usually manifesting in newborns or infants, 2) failure to thrive and dyslipidemia caused by citrin deficiency (FTTDCD), beyond age 1 year and 3) citrullinemia type II (CTLN2) with usually sudden manifestation between ages 20 and 50 years [2]. Children with NICCD often have a history of low birth weight with growth retardation. The disease is clinically characterised by intrahepatic cholestasis, hepatomegaly, diffuse fatty liver, variable liver dysfunction, hypoproteinemia, coagulopathy due to impaired hepatic synthesis of coagulation factors, haemolytic anemia and hypoglycemia [2, 11, 12]. NICCD is generally not severe, however, few patients required liver transplantation [13, 14], and fatal cases have been reported [15]. Many patients display markedly elevated galactose levels at the age of 1 month and the use of lactose-free milk should be considered in patients with hypergalactosemia [16]. Dietetic treatment with use of medium-chain triglyceride- (MCT-) enriched formulas has also been recommended [2, 16]. Interestingly, children with CD show a strong preference for protein-rich and lipid-rich foods and a natural aversion to sugar- and carbohydrate-rich foods [4, 16, 17]. Some patients with NICCD develop FTTDCD beyond the neonatal period and/or citrullinemia type II in adulthood, while the majority remains asymptomatic in later life [18]. Herein we report on two siblings, of whom the first was diagnosed with typical symptoms at the age of 8 weeks, while the younger brother was diagnosed immediately after birth. Both children responded well to dietary treatment, and patient 2 even remained asymptomatic.

## Case presentations

### Patient 1

The patient is the second child of non-consanguineous parents. The father is of Vietnamese origin, the mother of Romanian ancestry. The 2-year-old sister is healthy. The girl was born at 38 gestational weeks after an uneventful pregnancy. Her birth weight was 2770 g, birth length 48 cm, and head circumference 32 cm, Apgar 10/10. The family was discharged from hospital on day 4, but the girl presented again on day 8 with neonatal icterus (total bilirubin 21.2 mg/dl, direct bilirubin 0.8 mg/dl) requiring phototherapy. Apart from hyperbilirubinemia the clinical condition was very good, and the girl was fully breastfed. Phototherapy could be terminated after 23 h and bilirubin levels remained stable thereafter.

At the age of 8 weeks the child presented to the local pediatrician with a mild febrile airway infection. The girl was severely icteric but otherwise still in good clinical condition. The mother reported that the child required feeding every other hour, also at night. No stool or urine abnormalities were observed. She was admitted to the hospital for further diagnostic work-up. Laboratory testing revealed hepatopathy with the following parameters: prothrombin time 40%, INR 1.64, partial thromboplastin time 41 s., fibrinogen 63 mg/dL (normal 130–330 mg/dL), antithrombin 35%, AST 86 U/L (normal 10–35 U/L), ALT 28 U/L (normal 10–35 U/L), alkaline phosphatase 1367 U/L (normal < 449 U/L), gamma-GT 149 U/L (8–178 U/L), total bilirubin 10.2 mg/dL (normal < 1 mg/dL), direct bilirubin 4.9 mg/dL (normal < 0.3 mg/dL), total protein 4.0 g/dL (normal 4.4–7.6 g/dL), albumin 2.7 g/dL (normal 3.8–5.4 g/dL), alpha-fetoprotein > 12,000 ng/mL (normal < 77 ng/mL), and ferritin 1402 ng/mL (normal 15–150 ng/mL). The blood count was normal, and there were no signs of infection (CRP 3.4 mg/dL, normal < 3 mg/dL). Blood gas analysis yielded an elevated lactate concentration of 6.1 mmol/L. Ammonia concentration was 123 μmol/L (normal < 70 μmol/L).

Abdominal ultrasound showed liver size at the upper normal limit with enhanced echogenicity. No splenomegaly was detected, and the gall bladder as well as the bile ducts were normal. Polymerase chain reaction (PCR) analyses for CMV, enterovirus and parechovirus were negative, and toxoplasmosis was excluded serologically. Immunological investigations were not suggestive for immunological causes of the hepatopathy. Elastase concentration in stool was normal, and newborn screening for cystic fibrosis was unremarkable. Ultrasound of the brain, electrocardiogram and echocardiography were unremarkable.

During the following days the child displayed recurrent episodes of asymptomatic severe hypoglycemia with minimal glucose concentrations of 1.3 mmol/L despite frequent feeding (every 2 h). Lactate concentrations ranged from 0.9 to 6.1 mmol/L.

Metabolic investigations were interpreted as not suggestive of a specific disorder. Organic acids in urine showed massive tyrosyluria, well compatible with hepatopathy. Acylcarnitine analysis in dried blood spots yielded an elevated concentration of free carnitine with unspecifically elevated levels of several acylcarnitines. Amino acid analysis showed elevated concentrations of several amino acids including citrulline (331 μmol/L, normal < 35 μmol/L). The sialotransferrin pattern (screening for congenital disorders of glycosylation) was normal.

Due to the trias of hepatopathy/impaired hepatic function, lactic acidosis and recurrent hypoglycemia a hepatic form of a mitochondrial depletion syndrome was initially suspected, especially in combination with the markedly elevated concentrations of alpha fetoprotein and ferritin. Clinically, no neurologic involvement was observed, and the brain MRI showed unremarkable results. To prevent hypoglycemia breast milk was supplemented with MCT oil and maltodextrin. After 2 weeks, the child had to be transferred to the intensive care unit due to severe recurrent hypoglycemia. In the end, normal blood glucose levels could only be achieved by continuous i.v. glucose infusion or continuous oral feeding. Therefore, a percutaneous endoscopic gastrostomy (PEG) tube was inserted to enable normoglycemia and discharge from the hospital. Due to major PEG tube complications and a suspected intestinal perforation followed by a systemic infection inpatient treatment was prolonged. Lactate levels during this episode increased to 17 mmol/L. In the meanwhile, results of trio exome sequencing became available and revealed two mutations in the *SLC25A13* gene, one frameshift variant c.852_855del, p.Met285Profs*2 and a novel deletion c.(69 + 1_70–1)_(212 + 1_231–1)del, p.?. The father was found heterozygous for the frameshift variant by Sanger sequencing, whereas the mother is a carrier of the deletion, confirmed by qPCR.

**Fig. 1:**
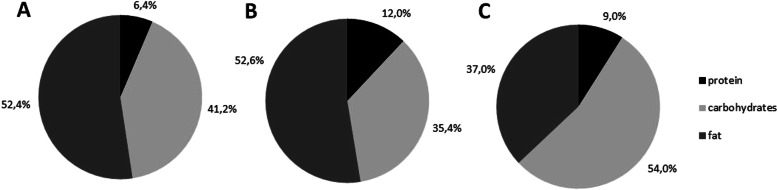
Comparison of relative nutrient intakes (proportion of total energy intake). **a** Average intake of a fully breast-fed child (**b**) Recommendation for our patient since diagnosis of CD. 35–46% of the fat intake was provided as MCT fat. **c** Average nutrient intake of a 1-year-old healthy child as recommended by the German Nutrition Society (DGE)

Since several attempts to implement tube-feeding with tea or small amounts of breast milk resulted in severe deterioration of abdominal symptoms, the PEG tube was removed after 3 weeks. Explorative laparotomy revealed extensive adhesions of the small intestine but no perforation. After removal of the PEG tube, the clinical condition stabilised and oral feeding could successfully be reintroduced. The child received a galactose-free, carbohydrate reduced, MCT-enriched diet consisting of Pregomin Proexpert (Milupa) and Basic–ch (Nutricia) (composition of nutrients displayed in Fig. 1). Under this regimen blood glucose levels remained stable with feeding intervals of 4 h. Hepatopathy and cholestasis resolved apart from a persistent mild elevation of transaminase activities. Lactate levels also normalised. At the age of 4 months the patient could be discharged from hospital in good clinical condition. Blood glucose monitoring at home revealed no further hypoglycemia.

**Fig. 2:**
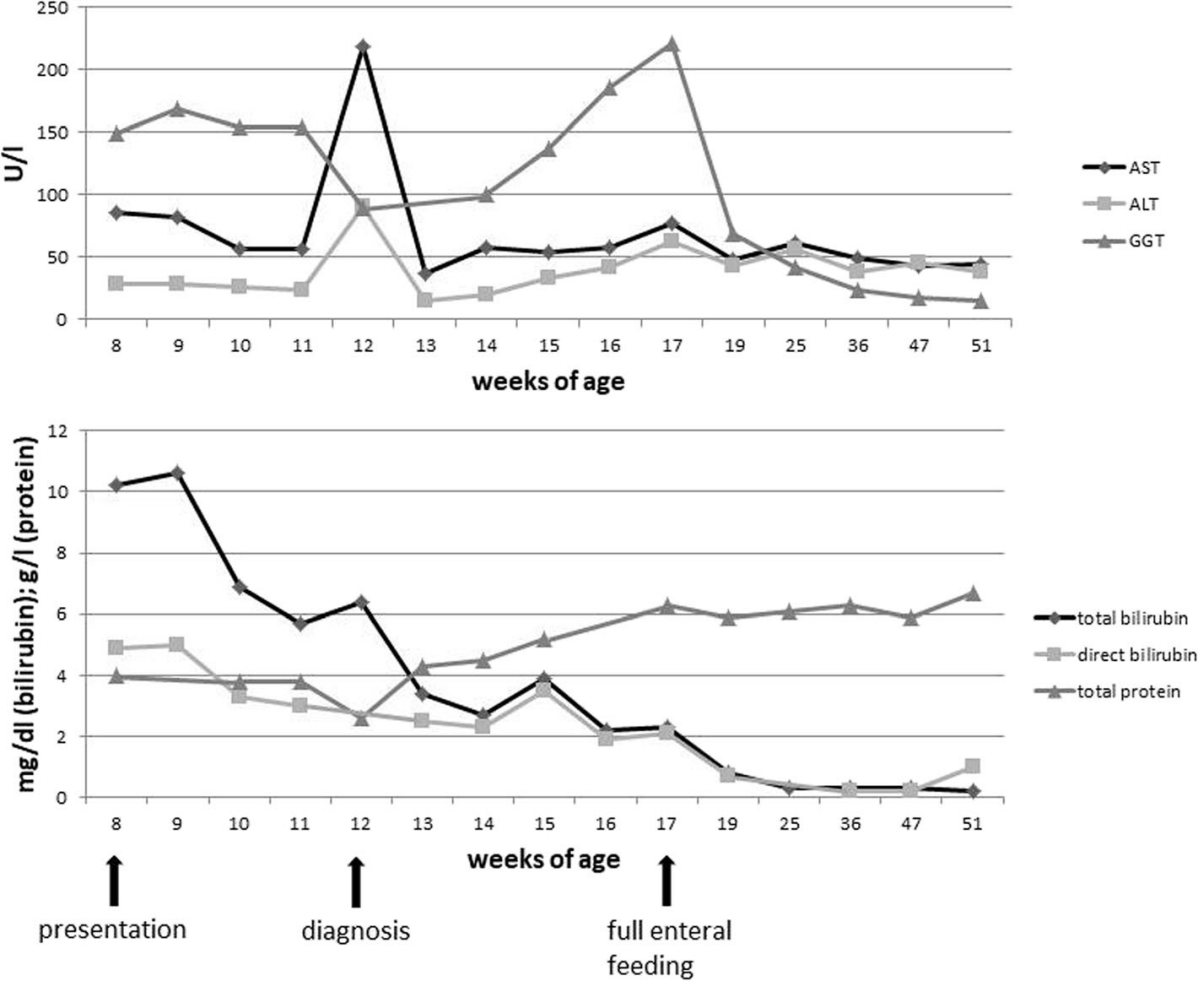
Laboratory parameters in the clinical course of patient 1 from presentation (8 weeks) until the age of 1 year

At the age of 6 months, supplementary foods were started without any complications. The diet was still galactose-free, carbohydrate-reduced and rich in protein and MCT. The girl is now 2 years old and shows normal psychomotor development. The only pathological findings are slightly elevated transaminase activities. Relevant laboratory parameters during the course of the disease are shown in Fig. 2.

### Patient 2

**Fig. 3:**
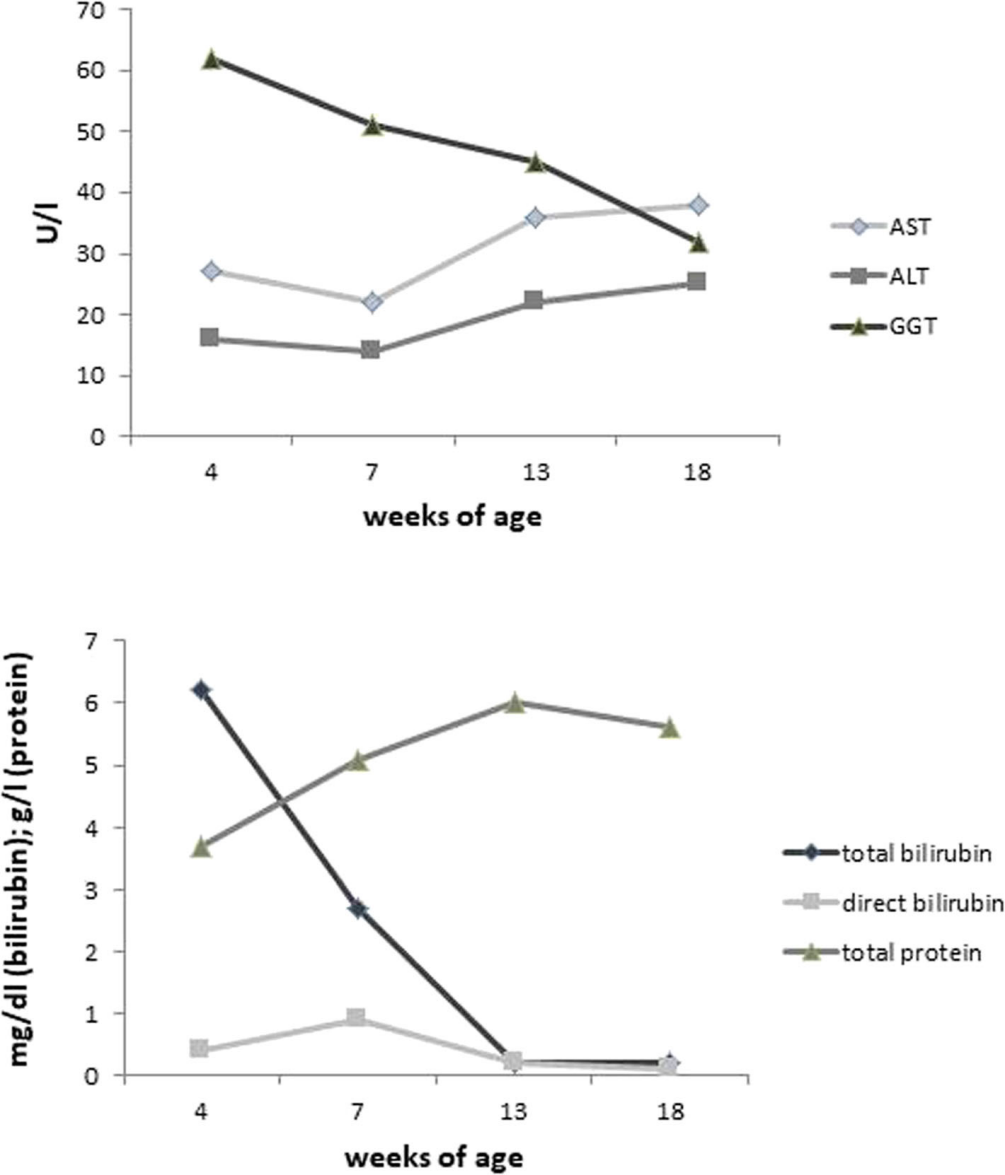
Laboratory parameters in the clinical course of patient 2 from birth until the age of 18 weeks

One year later her younger brother was born. Genetic testing was initiated immediately after birth. Until the results became available, a diet consisting of 50% breast milk and 50% Pregomin Proexpert (lactose-free, 50% MCT fat) was recommended. On day 12, CD was genetically confirmed by Sanger sequencing for the paternal mutation and qPCR for the maternal mutation. A lactose-free, MCT-enriched diet was started on the same day. He clinically remained asymptomatic with no signs of cholestasis or icterus. Laboratory monitoring was performed at age 4, 7, 13 and 18 weeks. Results of liver enzymes, bilirubin and total protein concentrations are displayed in Fig. 3.

### Newborn screening

CD is not a target disease of newborn screening programs in Germany. Nevertheless, citrulline levels as well as the levels of other diagnostic amino acids are measured in dried blood spots. Results of the amino acids measured by tandem mass spectrometry in the dried blood spots of the newborn screening samples of our two patients are displayed in Table 1.

**Table 1:** Relevant amino acid results measured by tandem mass spectrometry in dried blood spots newborn screening samples of both patients

Parameter	Patient 1	Patient 2	Reference range (μmol/L)
citrulline (μmol/L)	49	51	6–91.63
tyrosine (μmol/L)	49	32	< 350
phenylalanine (μmol/L)	38	32	< 123
alanine/citrulline ratio	3.18	3.86	n.a.

## Discussion and conclusions

We report on 2 siblings with compound heterozygosity for two variants in the *SLC25A13* gene of whom the index patient presented with cholestasis at age 8 weeks while in the younger brother clinical symptoms were possibly prevented by early initiation of a specific diet. The main findings in our patient apart from hepatopathy were severe recurrent hypoglycemia and elevated lactate concentrations. Both hypoglycemia and hyperlactatemia are no common biochemical abnormalities in patients with NICCD [11], although hypoglycemia has been reported in few patients [12, 16, 18, 19]. The pathophysiology of hypoglycemia in CD is not well understood. Young children are prone to develop hypoglycemia relatively easily due to CD-related suppression of gluconeogenesis [20]. It has additionally been suggested that severe hypoglycemia in patients with CD could be associated with relatively low levels of ketone bodies, implying that β-oxidation and ketogenesis in these patients might possibly be partially disrupted [19] due to altered redox states and PPAR dysregulation. The diagnosis of CD in our patient 1 was slightly delayed as the clinical picture was primarily suggestive of a hepatic form of a mitochondrial depletion syndrome, in particular DGUOK deficiency due to the markedly elevated levels of alpha fetoprotein and ferritin [21], and diagnosis was finally made by whole exome sequencing. Of the two mutations found in our patient, the c.852_855del, p.Met285Profs*2 variant is very common in Asian populations and derived from the father who was of Vietnamese origin [19, 22–24].

The second mutation, c.(69 + 1_70–1)_(212 + 1_231–1)del, has not been described previously, and the consequences of this variant on protein level are not known. However, since the deletion encompasses exon 3 of the *SLC25A13* gene, it is well conceivable that this variant is deleterious. In Europe, CD is an extremely rare inborn error of metabolism. However, globalisation and the concomitant worldwide spread of Asian populations will increase the likelihood of identifying more cases of NICCD outside the classical distribution area for CD [25]. This highlights the importance for pediatricians and metabolic physicians of being aware of CD as a differential diagnosis of neonatal cholestasis.

NICCD usually responds very well to dietary treatment and clinical symptoms resolve within months. This was also true for our patient 1 who showed an immediate response to dietary adaptations with resolution of hypoglycaemia and cholestasis. Nevertheless, few severe (requiring liver transplantation) or even fatal cases have been reported [13–15, 26, 27]. Abuduxikuer et al. investigated a cohort of 61 confirmed NICCD cases for risk factors associated with mortality [27]. Comparing 52 cases in the survival group with 9 fatal cases of NICCD the authors identified late referral, presence of infection, delayed treatment with lactose-free/MCT formula, lower platelet count, lower levels of gamma-GT, total cholesterol and blood citrulline and higher blood concentrations of ammonia and tyrosine as factors associated with poor prognosis [27].

Most patients with CD show a particular food preference from early childhood with a natural aversion against carbohydrate-rich foods and a strong preference for protein- and fat-rich foods, which is very different from the well-known aversion to protein among patients with other urea cycle defects [1, 16]. Saheki et al. studied the food intake of 18 Japanese citrin-deficient subjects with an age range from 1 to 33 years [4]. They found that the average relative fat and protein intake was higher in CD patients compared with published values for the general Japanese population with 134 and 116%, respectively. In contrast, the average relative carbohydrate intake was only 56% of age- and sex-matched Japanese controls. Carbohydrate, fat and protein provided 37, 44 and 19% of total energy in CD patients compared to 56, 27.5 and 14.5% in the healthy population. The peculiar food preferences may start from as early as 1 year of age, but they can take some time to be recognized by caregivers [4]. Nevertheless, food preferences may be a diagnostic hint for CD, even in the absence of other signs and symptoms [4, 19] and should prompt clinicians to rule out CD [4, 19].

Studies in animal models of CD have provided insights into possible mechanisms for reduced carbohydrate intake. Double knockout mice for mitochondrial glycerol-3- phosphate dehydrogenase and citrin, a phenotypic model for human CD, showed oral aversion to dietary sugar, ethanol and glycerol that correlated with alterations in specific hepatic metabolites [28]. The authors conclude that the aversion observed in the double-KO mice is mediated by hepatic metabolic perturbations, resulting in a behavioral response to increased hepatic cytosolic NADH and a decreased cellular adenine nucleotide pool [28]. The same findings may underlie the dietary predilections observed in CD patients. Under normal conditions, an increase in cytosolic NADH/NAD+ ratio following a carbohydrate-rich meal is relieved by NADH shuttle activity via the malate-aspartate and/or glycerol phosphate shuttles. Saheki et al. postulated that in CD patients the citrin defect leads to inhibition of ureagenesis by limiting the supply of aspartate for the urea cycle. This is not only due to the lack of aspartate supplied from mitochondria, but also because a high cytosolic NADH/NAD+ ratio reduces the cytosolic oxaloacetate concentration resulting in a shortage of cystosolic aspartate [4]. By reducing the intake of carbohydrates, CD patients can effectively minimize this deficiency of aspartate [4]. These pathophysiological considerations explain why some of the conventional treatment procedures for urea cycle defects/hyperammonemia may be very harmful in individuals with CD. A low-protein carbohydrate-rich diet is the standard therapy of urea cycle defects. However, if given to a patient with CD, this may cause severe metabolic disturbances such as hyperammonemia and hyperlipidemia [18, 29]. Similarly, high glucose or glycerol infusions (i.e. for brain edema) may result in severe clinical deterioration or even death in patients with CTNL2 [30–32]. Mutoh et al. reported that some Japanese children with CD have become severely ill after the entrance into primary school, where all school children had to take a high-carbohydrate lunch provided by the school [33]. These examples underline the importance of caregivers to respect the CD-typical food preferences to avoid clinical decompensations.

Tandem mass spectrometry-based newborn screening for CD is performed in certain countries with a high incidence of CD [34, 35]. Elevated citrulline levels and several citrulline-based ratios are the primary screening markers. However, it is known that citrulline levels in children with NICCD may not be elevated immediately after birth and several cases missed by newborn screening have been reported [13, 19, 36]. In a recent study with 55 patients with genetically proven NICCD, only 18 cases (18/55, 32.7%) were true positives and 37 cases (37/55, 67.3%) were false negatives based on the cut off value for citrulline in dried blood spots in newborn screening [35]. Blood sampling time seems to have an influence on the sensitivity of NICCD newborn screening, and false negative results were more common in the group of very early-screened patients. The citrulline levels in the newborn screening samples of our two patients were also normal. However, as CD is not a target disease of German newborn screening programs, cut off values established for the diagnosis of citrullinemia type 1 were used in our patients (displayed in Table 1) [37]. Clinical laboratory studies from countries with high incidence of CD have established lower cut-off values for citrulline in newborn screening [34, 38]. To improve the detection rate of NICCD in newborn screening the combined use of low citrulline cut offs together with second tier genetic screening has been suggested as the optimal strategy [34].

Since the treatment of citrullinemia type 1 and CD are very different and a protein-reduced, carbohydrate-enriched diet as suggested in citrullinemia type 1 aggravates symptoms in CD, a clear discrimination is essential directly after the newborn screening results have become available. This is sometimes challenging since mild forms of citrullinemia type 1 may only present with elevated citrulline without elevation of glutamine.

As CD is a well-treatable disorder this metabolic defect should be ruled out early in every infant with hepatic cholestasis and should also be considered in screened patients with mild elevations of citrulline. It is important to recognize that hypoglycemia can be part of the biochemical phenotype and that the clinical presentation may mimic hepatic mitochondrial depletion syndrome.

## Data Availability

Not applicable.

## Abbreviations

CD: Citrin deficiency
NICCD: Neonatal intrahepatic cholestasis caused by citrin deficiency
MCT: Middle-chain triglycerides
PCR: Polymerase chain reaction
PEG: Percutaneous endoscopic gastrostomy

## References

[1] Saheki T, Inoue K, Tushima A, Mutoh K, Kobayashi K (2010). Citrin deficiency and current treatment concepts. Mol Genet Metab.

[2] Saheki T, Song Y-Z, Adam MP, Ardinger HH, Pagon RA, Wallace SE, Bean LJ, Stephens K (1993). Citrin deficiency. GeneReviews®.

[3] Saheki T, Kobayashi K (2002). Mitochondrial aspartate glutamate carrier (citrin) deficiency as the cause of adult-onset type II citrullinemia (CTLN2) and idiopathic neonatal hepatitis (NICCD). J Hum Genet.

[4] Saheki T, Kobayashi K, Terashi M, Ohura T, Yanagawa Y, Okano Y (2008). Reduced carbohydrate intake in citrin-deficient subjects. J Inherit Metab Dis.

[5] Palmieri L, Pardo B, Lasorsa FM, del Arco A, Kobayashi K, Iijima M (2001). Citrin and aralar1 are Ca(2+)-stimulated aspartate/glutamate transporters in mitochondria. EMBO J.

[6] Dimmock D, Maranda B, Dionisi-Vici C, Wang J, Kleppe S, Fiermonte G (2009). Citrin deficiency, a perplexing global disorder. Mol Genet Metab.

[7] Dimmock D, Kobayashi K, Iijima M, Tabata A, Wong L-J, Saheki T (2007). Citrin deficiency: a novel cause of failure to thrive that responds to a high-protein, low-carbohydrate diet. Pediatrics..

[8] Ben-Shalom E, Kobayashi K, Shaag A, Yasuda T, Gao H-Z, Saheki T (2002). Infantile citrullinemia caused by citrin deficiency with increased dibasic amino acids. Mol Genet Metab.

[9] Hutchin T, Preece MA, Hendriksz C, Chakrapani A, McClelland V, Okumura F (2009). Neonatal intrahepatic cholestasis caused by citrin deficiency (NICCD) as a cause of liver disease in infants in the UK. J Inherit Metab Dis.

[10] Fiermonte G, Soon D, Chaudhuri A, Paradies E, Lee PJ, Krywawych S (2008). An adult with type 2 citrullinemia presenting in Europe. N Engl J Med.

[11] Wang J-S, Wang X-H, Zheng Y-J, Fu H-Y, Chen R, Lu Y (2012). Biochemical characteristics of neonatal cholestasis induced by citrin deficiency. World J Gastroenterol.

[12] Ohura T, Kobayashi K, Tazawa Y, Abukawa D, Sakamoto O, Tsuchiya S (2007). Clinical pictures of 75 patients with neonatal intrahepatic cholestasis caused by citrin deficiency (NICCD). J Inherit Metab Dis.

[13] Tamamori A, Okano Y, Ozaki H, Fujimoto A, Kajiwara M, Fukuda K (2002). Neonatal intrahepatic cholestasis caused by citrin deficiency: severe hepatic dysfunction in an infant requiring liver transplantation. Eur J Pediatr.

[14] Shigeta T, Kasahara M, Kimura T, Fukuda A, Sasaki K, Arai K (2010). Liver transplantation for an infant with neonatal intrahepatic cholestasis caused by citrin deficiency using heterozygote living donor. Pediatr Transplant.

[15] Zhao X-J, Tang X-M, Zha Q-B, Shi S-S, Song Y-Z, Xiao X-M (2011). Prenatal diagnosis of citrin deficiency in a Chinese family with a fatal proband. Tohoku J Exp Med.

[16] Okano Y, Ohura T, Sakamoto O, Inui A (2019). Current treatment for citrin deficiency during NICCD and adaptation/compensation stages: strategy to prevent CTLN2. Mol Genet Metab.

[17] Hachisu M, Oda Y, Goto M, Kobayashi K, Saheki T, Ohura T (2005). Citrin deficiency presenting with ketotic hypoglycaemia and hepatomegaly in childhood. Eur J Pediatr.

[18] Saheki T, Kobayashi K, Iijima M, Moriyama M, Yazaki M, Takei Y-I (2005). Metabolic derangements in deficiency of citrin, a liver-type mitochondrial aspartate-glutamate carrier. Hepatol Res.

[19] Chong SC, Lo P, Chow CW, Yuen L, Chu WCW, Leung TY (2018). Molecular and clinical characterization of citrin deficiency in a cohort of Chinese patients in Hong Kong. Mol Genet Metab Rep.

[20] Wada Y, Arai-Ichinoi N, Kikuchi A, Sakamoto O, Kure S (2020). Hypoketotic hypoglycemia in citrin deficiency: a case report. BMC Pediatr.

[21] Freisinger P, Fütterer N, Lankes E, Gempel K, Berger TM, Spalinger J (2006). Hepatocerebral mitochondrial DNA depletion syndrome caused by deoxyguanosine kinase (DGUOK) mutations. Arch Neurol.

[22] Song Y-Z, Deng M, Chen F-P, Wen F, Guo L, Cao S-L (2011). Genotypic and phenotypic features of citrin deficiency: five-year experience in a Chinese pediatric center. Int J Mol Med.

[23] Lin W-X, Zeng H-S, Zhang Z-H, Mao M, Zheng Q-Q, Zhao S-T (2016). Molecular diagnosis of pediatric patients with citrin deficiency in China: SLC25A13 mutation spectrum and the geographic distribution. Sci Rep.

[24] Fu H-Y, Zhang S-R, Wang X-H, Saheki T, Kobayashi K, Wang J-S (2011). The mutation spectrum of the SLC25A13 gene in Chinese infants with intrahepatic cholestasis and aminoacidemia. J Gastroenterol.

[25] Vitoria I, Dalmau J, Ribes C, Rausell D, García AM, López-Montiel J (2013). Citrin deficiency in a Romanian child living in Spain highlights the worldwide distribution of this defect and illustrates the value of nutritional therapy. Mol Genet Metab.

[26] Zhang Z-H, Yang Z-G, Chen F-P, Kikuchi A, Liu Z-H, Kuang L-Z (2014). Screening for five prevalent mutations of SLC25A13 gene in Guangdong, China: a molecular epidemiologic survey of citrin deficiency. Tohoku J Exp Med.

[27] Abuduxikuer K, Chen R, Wang Z-L, Wang J-S (2019). Risk factors associated with mortality in neonatal intrahepatic cholestasis caused by citrin deficiency (NICCD) and clinical implications. BMC Pediatr.

[28] Saheki T, Inoue K, Ono H, Fujimoto Y, Furuie S, Yamamura K-I (2017). Oral aversion to dietary sugar, ethanol and glycerol correlates with alterations in specific hepatic metabolites in a mouse model of human citrin deficiency. Mol Genet Metab.

[29] Imamura Y, Kobayashi K, Shibatou T, Aburada S, Tahara K, Kubozono O (2003). Effectiveness of carbohydrate-restricted diet and arginine granules therapy for adult-onset type II citrullinemia: a case report of siblings showing homozygous SLC25A13 mutation with and without the disease. Hepatol Res.

[30] Takahashi H, Kagawa T, Kobayashi K, Hirabayashi H, Yui M, Begum L (2006). A case of adult-onset type II citrullinemia--deterioration of clinical course after infusion of hyperosmotic and high sugar solutions. Med Sci Monit.

[31] Yazaki M, Takei Y, Kobayashi K, Saheki T, Ikeda S (2005). Risk of worsened encephalopathy after intravenous glycerol therapy in patients with adult-onset type II citrullinemia (CTLN2). Intern Med.

[32] Fukushima K, Yazaki M, Nakamura M, Tanaka N, Kobayashi K, Saheki T (2010). Conventional diet therapy for hyperammonemia is risky in the treatment of hepatic encephalopathy associated with citrin deficiency. Intern Med.

[33] Mutoh K, Kurokawa K, Kobayashi K, Saheki T (2008). Treatment of a citrin-deficient patient at the early stage of adult-onset type II citrullinaemia with arginine and sodium pyruvate. J Inherit Metab Dis.

[34] Lin Y, Liu Y, Zhu L, Le K, Shen Y, Yang C (2019). Combining newborn metabolic and genetic screening for neonatal intrahepatic cholestasis caused by citrin deficiency. J Inherit Metab Dis.

[35] Tang CF, Liu SC, Feng Y, Mei HF, Liu HP, Feng JW (2019). Newborn screening program and blood amino acid profiling in early neonates with citrin deficiency. Zhonghua Er Ke Za Zhi.

[36] Estrella J, Wilcken B, Carpenter K, Bhattacharya K, Tchan M, Wiley V (2014). Expanded newborn screening in New South Wales: missed cases. J Inherit Metab Dis.

[37] Sander J, Janzen N, Sander S, Steuerwald U, Das AM, Scholl S (2003). Neonatal screening for citrullinaemia. Eur J Pediatr.

[38] Tamamori A, Fujimoto A, Okano Y, Kobayashi K, Saheki T, Tagami Y (2004). Effects of citrin deficiency in the perinatal period: feasibility of newborn mass screening for citrin deficiency. Pediatr Res.

